# Frequency of HIV Infection among Sailors in South of Iran by Rapid HIV Test

**DOI:** 10.1155/2011/612475

**Published:** 2011-04-10

**Authors:** Hossain Jabbari, Somayeh Aghamollaie, Gholamreza Esmaeeli Djavid, Abbas Sedaghat, Maryam Sargolzaei, SeyedAhmad SeyedAlinaghi, Mehrnaz Rasoolinejad, Minoo Mohraz

**Affiliations:** ^1^Iranian Research Center for HIV/AIDS (IRCHA), Tehran University of Medical Sciences, Tehran, Iran; ^2^Academic Center for Education, Culture and Research, Tehran, Iran; ^3^Ministry of Health and Medical Education, Tehran, Iran

## Abstract

Information on the prevalence and risk factors for HIV infection among sailors is scarce. The aim of this seroprevalence study was to evaluate the frequency of HIV infection among sailors in south of Iran using rapid HIV test. The study included 400 consecutive participants in Lengeh, Shahid Rajaie, and Shahid Bahonar ports in south of Iran in May 2010. We observed only one case (0.25%) of HIV infection in this sample of sailors. While prevalence appears low at present, we recommend periodic HIV serosurveillance with detailed behavioral measures for this population in the future.

## 1. Introduction

According to WHO protocol sailors, truck drivers and immigrant workers are low-risk population for HIV infection. Truck drivers and immigrant workers were studied in Iran [1, 2] while information on the prevalence and risk factors for HIV infection among sailors is generally scarce. Some studies have shown that sailors who arrived from abroad and spent at least one night in ports and most of their time traveling in different countries far from their family and sexual partners are at high risk of HIV infection through high risk sexual contacts with sex workers and have been used as a sentinel population in surveillance programs [3, 4].

 To our knowledge, no similar study has been conducted among this population in Iran. This study assessed the frequency of HIV infection among sailors in south of Iran as a sentinel population for HIV/AIDS using the rapid HIV test. 

## 2. Materials and Methods

In this cross-sectional study, we approached 409 consecutive Hormozgan Province's sailors from the 1st to the 31st of May 2010. Of these, 400 participants consented to attend anonymously. This study was approved by institutional review board (IRB) of Tehran University of Medical Sciences (TUMS) and was done in Shahid Rajaie, Lengeh, and Shahid Bahonar ports located in south of Iran. The sailors were traveling to Asian, European, and African countries, and they were approached while waiting at the harbors. Basic demographic information (age and marital status) was recorded in data collection sheet. After obtaining verbal consent, 40 microliter of blood was collected by sampler for rapid HIV test (Acon Laboratories, Inc., San Diego, CA 92121, USA). Sensitivity and specificity of the test were 99.9% and 99.8%, respectively. The test results were read after 3–5 minutes and analyzed by SPSS Software. People whose tests were initially screened as positive or indeterminate were referred to the Imam Khomeini Hospital (the national referral center for HIV/AIDS) in Tehran for confirmation and further care.

## 3. Results

Among the sailors surveyed, the mean age was 39.34 ± 12/068 years (range, 18–80 years). 331 people were married (82.8%), 67 single (16.8%), and 2 divorced (0.4%). We found only one (0.25%, %95 CI %0.006–%1.38) HIV positive test among this sample of Iranian sailors in the south ports. After referring to the hospital, ELIZA and Western blot tests of the individual were positive. He had history of several trips to Pakistan and India.

## 4. Discussion

In our study, we found only one HIV positive test. Possible reasons for our results include the following. (1) There is truly very little HIV infection in Iranian sailors. (2) Our sample represents lower risk sailors compared to samples of sailors in other countries.  Table 1 shows seroprevalence of HIV infection among other countries. Our study shows the similar statistics to some countries such as UK and West Africa. Also it shows the significant difference with Ethiopia and Spain.

Our major limitation of our study is that we did not ask about risk behaviors in this sample. Despite this limitation, our study provides the first estimate of HIV seroprevalence among sailors in Iran. Regarding similar studies in other countries, periodical tests in this sentinel population along with necessary training in order to improve insight, knowledge, and preventive measures about HIV/AIDS appear to be essential. We propose future studies to evaluate behavioral risk factors in sailors regarding paucity of data in this population in Iran. Finally, we also suggest expanding similar studies at other harbors which may attend more high risk sailors.

## Figures and Tables

**Table 1:** Prevalence of HIV infection among sailors in other countries.

Country	Sample size	Prevalence
Ethiopia [5]	260	9.6%
Spain [6]	57	3.5%
UK [7]	304	0.33%
Sub-Saharan WestAfrica [8]	499	0.2%
